# A marginal structural model to estimate the effect of antidepressant medication treatment on major cardiovascular events among people with post-traumatic stress disorder

**DOI:** 10.1017/S0033291723001873

**Published:** 2023-12

**Authors:** Kwanghyun Kim, Sunghyuk Kang, Chung Mo Nam, Robert Stewart, Alexander C. Tsai, Sun Jae Jung

**Affiliations:** 1 Department of Preventive Medicine, Yonsei University College of Medicine, Seoul, Korea; 2 Department of Public Health, Graduate School, Yonsei University, Seoul, Korea; 3 Department of International Health, Johns Hopkins Bloomberg School of Public Health, Baltimore, Maryland, USA; 4 Center for Humanitarian Health, Johns Hopkins Bloomberg School of Public Health, Baltimore, Maryland, USA; 5 King's College London (Institute of Psychiatry, Psychology and Neuroscience), London, UK; 6 South London and Maudsley NHS Foundation Trust, London, UK; 7 Center for Global Health, Massachusetts General Hospital, Boston, Massachusetts, USA; 8 Harvard Medical School, Boston, Massachusetts, USA; 9 Harvard Center for Population and Development Studies, Cambridge, Massachusetts, USA

**Keywords:** cardiovascular disease, cohort study, inverse probability of treatment weighting, marginal structural model, Posttraumatic stress disorder

## Abstract

**Background:**

Previous evidence on antidepressant medication and cardiovascular disease (CVD) among patients with posttraumatic stress disorder (PTSD) has been inconclusive. We estimated the association between antidepressant medication and CVD by applying a marginal structural model.

**Methods:**

We analyzed medical utilization records of 27 170 people with PTSD without prior major cardiovascular events in the Korean National Health Insurance Database (NHID). PTSD and CVD were defined in accordance with the recorded ICD-10 diagnostic codes. We acquired information on antidepressant use from the NHID and categorized them by medication type. A composite major adverse cardiovascular events (MACE) outcome was defined as coronary artery disease with revascularization, ischaemic stroke, and/or haemorrhagic stroke. We used inverse probability of treatment weighting to estimate the parameters of a marginal structural discrete-time survival analysis regression model, comparing the resulting estimates to those derived from traditional time-fixed and time-varying Cox proportional hazards regression. We calculated cumulative daily defined doses to test for a dose–response relationship.

**Results:**

People exposed to antidepressants showed a higher hazard of MACE [hazard ratio (HR) 1.34, 95% confidence interval (CI) 1.18–1.53]. The estimated effects were strongest for selective serotonin reuptake inhibitors (HR 1.24, 95% CI 1.08–1.44) and TCAs (HR 1.33, 95% CI 1.13–1.56). Exposure to serotonin-norepinephrine reuptake inhibitors did not appear to increase the risk of MACE. People exposed to higher doses of antidepressants showed higher risk of MACE.

**Conclusions:**

In a national cohort of people with PTSD, exposure to antidepressant medications increased the risk of MACE in a dose–response fashion.

## Introduction

Previous studies have suggested that patients with posttraumatic stress disorder (PTSD) have a higher risk of cardiovascular disease (CVD), including ischaemic heart disease, (Ebrahimi et al., 2021; Edmondson & Cohen, 2013) stroke, (Gaffey et al., 2021; Yu, Alper, Nguyen, Maqsood, & Brackbill, 2021) and cardiovascular mortality (Boscarino, 2008). PTSD has also been linked to hypertension (Gaffey et al., 2020) and diabetes, (Roberts et al., 2015) which are major risk factors for CVD. Depressive and anxiety disorders, which are often comorbid with PTSD, have also been implicated in the development of stroke (Zahodne et al., 2017) and coronary heart disease (Roest, Martens, de Jonge, & Denollet, 2010; Zahodne et al., 2017). These links between PTSD and CVD have been attributed to alterations in underlying neurohormonal systems, such as the hypothalamic‒pituitary‒adrenal axis and the sympathetic-adrenal-medullary systems, (Lima et al., 2019; Vaccarino & Bremner, 2017) and consequent inflammatory reactions (Lima et al., 2019; Michopoulos, Powers, Gillespie, Ressler, & Jovanovic, 2017).

Pharmacologic agents, primarily antidepressant medications, are often used among patients with PTSD, because they are effective in treating depressive disorders, which are highly comorbid with PTSD (Zahodne et al., 2017), and because they are effective in attenuating several characteristic symptoms of PTSD, including hyperarousal, avoidance, and re-experiencing (Pearlstein, 2000; Petrakis et al., 2012; Puetz, Youngstedt, & Herring, 2015). There have been attempts to assess the cardiovascular effects of antidepressant medication among patients with PTSD, but no definitive conclusions have been drawn from previous studies: on the one hand, alleviation of PTSD symptoms could blunt neurohormonal responses and ultimately mitigate the potentially cardiotoxic sequelae of PTSD (Gilsanz et al., 2017); on the other hand, a body of work has shown that antidepressant medications are associated with an increased risk of stroke (Gaffey et al., 2021; Glymour et al., 2019; Smoller et al., 2009), myocardial infarction (Cohen, Gibson, & Alderman, 2000; Grace et al., 2018), and cardiovascular mortality (Grace et al., 2018; He, Zhou, Ma, Wei, & Fu, 2020).

While the converging strands of literature cited previously suggest the importance of this line of enquiry, there is limited evidence on how antidepressant use can affect CVD risk among people with PTSD. A recent analysis using data from 143 324 women veterans in the U.S. showed that selective serotonin reuptake inhibitors (SSRIs) and serotonin-norepinephrine reuptake inhibitors (SNRIs) are associated with greater risk of ischaemic heart disease, emphasizing the potential cardiovascular toxicity of antidepressant medication among patients with PTSD (Ebrahimi et al., 2022). However, in a study of 1079 US veterans that used inverse probability of treatment (IPT) weighting to control for potential confounding, neither improvement in symptoms of PTSD nor antidepressant use were associated with incident CVD (Scherrer et al., 2020). These results stand in contrast to a previously published finding of increased inflammatory cytokine levels among people with PTSD exposed to antidepressant medications (Sumner et al., 2017).

**Figure 1.**
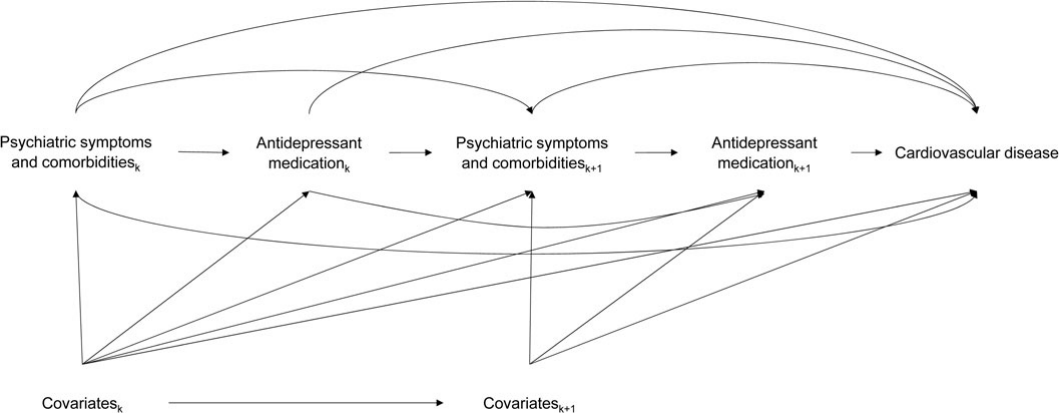
Directed acyclic graph describing concurrent associations between antidepressant medication use, psychiatric symptoms and comorbidities, and cardiovascular disease. Comorbidity and symptom severity act as time-varying confounders that also potentially mediate the association between antidepressant use and cardiovascular disease.

The mixed findings of this literature suggest that estimating the potential (positive or negative) cardiovascular effects of exposure to antidepressant medications is challenging. Complex relationships between PTSD symptoms, its psychiatric comorbidities, antidepressant use and CVD increase the complexity of effect estimation, which requires appropriate statistical models to disentangle the complex web of association. Psychiatric symptom severity affects the probability of being exposed to antidepressant medications while also confounding the association between antidepressant medication and CVD (Fig. 1). Patients with more severe symptoms and other psychiatric comorbidities are more likely to use antidepressant medications, so symptomatology and comorbidity should be appropriately adjusted for in the analysis (Glymour et al., 2019). Patients with more severe symptoms are more likely to be treated by other psychiatric medications such as benzodiazepines (Williams, Phillips, Stein, & Ipser, 2022) and second-generation antipsychotics (Abdallah et al., 2019), further complicating the estimation of the antidepressant-CVD association. Therefore, an analytic model that is capable of handling time-varying confounding factors affected by prior treatment is needed to estimate the potential causal relationships between antidepressant medications and CVD (Hernán, Brumback, & Robins, 2000; Robins, Hernan, & Brumback, 2000).

Given the limited evidence in this area, we analysed a nationwide database from South Korea to estimate the causal effects of antidepressant medication treatment on CVD outcomes among people with PTSD. To adjust for the potentially confounding effects of time-varying psychiatric symptoms and comorbidities influenced by prior antidepressant exposure, we used IPT weighting to estimate the parameters of a marginal structural discrete-time survival analysis regression model and compared the resulting estimates to those derived from conventional Cox regression models.

## Methods

### Patient enrolment

This study is a secondary analysis of data from the Korean National Health Insurance Database (NHID), which is an administrative database constructed by the National Health Insurance System (NHIS) of South Korea. The database consists of all medical service utilization covered by the NHIS, including all outpatient and inpatient medical services. Medical service utilization data of people with PTSD who received medical services during 2004–2018 were obtained from NHID. The database consists of all administrative records of medical service usage of approximately 98% of the South Korean population, with information on eligibility, demographic characteristics, diagnosis, and treatment (Kwon, 2009). Individuals with at least one ICD-10 diagnostic code for PTSD (F43.1) during 2004–2018 were categorized as having PTSD (*N* = 109 235), and the date of the first recorded PTSD diagnosis was set as the index date. Participants were excluded if (i) they were aged 18 years or younger at the index date, (ii) they were missing data on sociodemographic characteristics, (iii) they had never received a medical check-up from NHIS, (iv) they had a prior diagnosis of a major cardiovascular event (coronary artery disease with revascularization, ischaemic stroke, and/or haemorrhagic stroke) before the index date, and/or (v) they had received antidepressant medication treatment before the index date. As a result, data from 27 170 people with PTSD were used for the final analysis (online Supplementary Material 1).

### Exposures and outcomes

Data on antidepressant medication use were collected from the NHID claims data. Medications were classified as SSRIs, SNRIs, and tricyclic antidepressants (TCAs). The full list of antidepressant medications in the NHID is shown in online Supplementary Material 2. Information on the types and doses of antidepressant medication was obtained from the NHID medical service utilization record. We estimated the cumulative daily defined dose (DDD) of antidepressant medication treatment for each time interval, in accordance with the definition provided by the World Health Organization Collaborating Centre for Drug Statistics Methodology (WHO Collaborating Centre for Drug Statistics Methodology, 2013). Cumulative DDD at a specified time interval was defined as the sum of DDD of all antidepressant medications recorded. The cumulative DDD of antidepressant medications was updated every 3 months after enrolment.

We defined major adverse cardiovascular events (MACE) as a composite of incident coronary artery disease with revascularization, incident ischaemic stroke, incident haemorrhagic stroke, and cardiovascular mortality (Bosco, Hsueh, McConeghy, Gravenstein, & Saade, 2021; Sharma et al., 2020). People with one or more ICD-10 codes for myocardial infarction (I21) or one or more ICD-10 codes for other ischaemic heart diseases (I20, I24) with a subsequent revascularization procedure were categorized as having ‘coronary artery disease with revascularization’ within the same treatment session. People with one or more ICD-10 codes for ischaemic stroke (I63) or haemorrhagic stroke (I60, I61, I62) with a brain imaging study within the treatment session were defined as having ‘ischaemic stroke’ or ‘haemorrhagic stroke’, respectively. All mortality cases from CVDs designated by ICD-10 ‘I’ codes were defined as cardiovascular mortality. For all CVD outcomes, we excluded outpatient diagnoses and only included inpatient diagnoses and diagnoses in the emergency department. Participants were considered to be censored at the time point when any one of the four CVD outcomes listed above were diagnosed. For people without MACE, the date of the last insurance claim was set as the endpoint of follow-up. If participants received another CVD diagnosis but did not meet the criteria of MACE, they were not censored from the analyses and considered to be followed up unti thel first diagnosis of MACE or the date of the last insurance claim.

### Covariates

We collected information on CVD risk factors from the NHIS medical check-up database, which consists of health check-up data from NHIS subscribers. Body mass index, serum total cholesterol, fasting blood glucose, blood pressure, cigarette smoking, alcohol consumption age at index date and sex were included as covariates. Participants' current smoking status was categorized as ‘current smoker’ vs. ‘ex-smoker/non-smoker’. Frequency of alcohol consumption was categorized as follows: ‘almost never’, ‘twice ~ three times a month’, ‘once ~ twice a week’, ‘3–4 times a week’, and ‘daily or almost daily’. Participants who consumed alcoholic beverages ‘twice ~ three times a month’ or more frequently were defined as ‘current drinkers.’

People with ICD-10 diagnostic codes for psychiatric disorders were considered to have the corresponding symptoms and comorbidities. Psychotic disorders (F20, F21, F22, F23, F24, F25, F28, F29), manic episode/bipolar disorders (F30, F31), depressive disorders (F32, F33, F34), anxiety and related disorders (F40, F41, F42, F43) and somatoform disorders (F45) were included as symptoms to predict the probability of antidepressant medication use. Each ICD-10 code was included in the model as a separate covariate. Claim records with both ICD-10 codes for psychiatric disorders (‘F’ codes) and insurance claim codes for hospital admission were defined as psychiatric hospitalizations. The monthly insurance premium was also included as a covariate, given its widespread use as a surrogate variable of socioeconomic position in analysis of the NHID (Khang, Yang, Cho, Jung-Choi, & Yun, 2010; Song et al., 2006; Song & Byeon, 2000). All variables except for age at index date were measured as time-varying covariates, with covariate values updated every 3 months from the index date. If values for time-varying covariates were missing at a certain time interval, values from the previous time interval were carried forward.

### Statistical analysis

We compared characteristics of participants by history of exposure to antidepressant medication. For MACE, psychiatric comorbidities, and psychiatric hospitalizations, we compared numbers and proportions of participants at the end of follow-up. For other covariates, we compared baseline characteristics of participants. We used the *t* test for comparing continuous variables and the chi-square test for comparing categorical variables.

A directed acyclic graph describing our analytic framework is presented in Fig. 1. For each time interval *I*_*k*_ = [*t*_*k*_, *t*_*k*+1_], *A_k_*, *L_k_*, and *Y* indicate the status of antidepressant medication use, time-varying covariates, and censoring status at time interval *I*_*k*_, respectively. The causal relationship of interest is the effect of antidepressant medication treatment on cardiovascular outcomes. Potential confounding by psychiatric symptoms and comorbidities should be controlled because they influence both the probability of antidepressant medication exposure and the risk of MACE. However, antidepressant medication treatment also influences subsequent psychiatric symptoms and comorbidities. To adjust for this time-varying confounding affected by prior treatment, we used IPT weighting to estimate the parameters of a marginal structural model (Robins, 2000; Robins et al., 2000; Tsai et al., 2010). We calculated IPT weights for the main analysis and the dose–response analysis separately. The process of calculating the IPT weights and estimating the marginal structural model is presented in online Supplementary Material 3. Additionally, we used polynomial splining with degree of 5 to check for nonlinear associations, with knots at the 5p, 25p, 50p, 75p, and 95p of cumulative DDD.

We conducted a sensitivity analysis by progressively truncating the stabilized IPT weights (at the 1p and 99p, the 5p and 95p, and the 10p and 90p) to probe for possible violations of positivity (Cole & Hernán, 2008). Additionally, we estimated e-values to assess the extent to which an unmeasured confounder could potentially explain away the estimated association between antidepressant medication and incident MACE (VanderWeele & Ding, 2017). Finally, to account for potential differences in psychiatric comorbidities, we conduced a sensitivity analysis by excluding participants diagnosed with psychotic disorders, manic episodes/bipolar disorders, anxiety and related disorders, and somatoform disorders, respectively, and re-estimating the models.

## Results

### Baseline characteristics

There were 27 170 participants included in the analysis, followed for a mean of 7.97 years [standard deviation (s.d.) 4.41 years]. Of the people included in the analysis, 14 743 people received antidepressant medication after being diagnosed with PTSD. Compared to men, women were more likely to be exposed to antidepressant medications (***p* < 0.001**). The mean monthly insurance premium of participants who received antidepressant medications was lower than that of participants who did not (*p* < 0.001). The mean age of people exposed to antidepressant medication was 39.84 years (s.d. 12.53) at the index date, and the mean age of those who did not receive antidepressants was 39.76 years (s.d. 12.77; *p* = 0.080). The mean follow-up period was 7.45 years (s.d. 4.48) among people who received antidepressants and 8.61 years (s.d. 4.38) among those who did not (***p* < 0.001**). Coronary artery disease with revascularization (***p* < 0.001**) and ischaemic stroke (***p* < 0.001**) were more common among people exposed to antidepressant medications, but there were no statistically significant differences in haemorrhagic stroke (*p* = 0.680; Table 1).

**Table 1.** Baseline characteristics of participants by exposure to antidepressant medication (*N* = 27 170)

	Full cohort (*N* = 27 170)	Never exposed to antidepressant medication (*N* = 12 427)	Ever exposed to antidepressant medication (*N* = 14 743)	*p* value
**Age at index date, mean (s.d.)**	39.78 (12.30)	39.84 (12.53)	39.76 (12.76)	0.080
**Men, *N*** (**%)**	11 023 (40.57)	5500 (41.54)	5523 (39.65)	**<0.001**
**Monthly insurance premium, KRW, *N*** (**%)**				**<0.001**
0 (Medicaid recipients)	910 (3.35)	286 (2.30)	624 (4.23)	
⩽20p (1–15 300)	4721 (17.38)	2065 (16.62)	2656 (18.02)	
20–40p (15 301–27 300)	5787 (21.30)	2580 (20.76)	3207 (21.75)	
40–60p (27 301–41 000)	5357 (19.72)	2441 (19.64)	2916 (19.78)	
60–80p (41 001–59 300)	5394 (19.85)	2612 (21.02)	2782 (18.87)	
⩾80p (⩾59 311)	5017 (18.47)	2235 (17.99)	2782 (18.87)	
N/A	477 (1.76)	208 (1.67)	269 (1.82)	
**Body mass index, kg/m^2^, mean (s.d.)**	23.41 (3.31)	23.41 (3.27)	23.40 (3.35)	**0.006**
**Systolic blood pressure, mmHg, mean (s.d.)**	120.55 (15.94)	120.64 (16.01)	120.34 (15.83)	0.365
**Diastolic blood pressure, mmHg, mean (s.d.)**	75.67 (10.77)	75.72 (10.82)	75.53 (10.72)	0.364
**Total cholesterol, mg/dL, mean (s.d.)**	193.07 (42.55)	192.53 (45.51)	193.34 (38.16)	**<0.001**
**Fasting blood glucose, mg/dL, mean (s.d.)**	95.20 (26.02)	94.60 (24.01)	95.65 (27.70)	**<0.001**
**Current cigarette smoking, *N*** (**%)**	6360 (23.41)	2737 (22.02)	3623 (24.57)	**<0.001**
**Current alcohol consumption, *N*** (**%)**	16 097 (59.25)	7551 (60.76)	8546 (57.97)	**<0.001**
**Years of follow-up, mean (s.d.)**	7.97 (4.41)	8.61 (4.38)	7.45 (4.48)	**<0.001**
**Major cardiovascular events, *N*** (**%)**^a^				**<0.001**
CAD with revascularization	951 (3.50)	337 (2.71)	614 (4.16)	**<0.001**
Ischaemic stroke	1014 (3.73)	378 (3.04)	636 (4.31)	**<0.001**
Haemorrhagic stroke	535 (1.97)	240 (1.93)	295 (2.00)	0.680
**Psychiatric comorbidities, *N*** (**%)**^a^				**<0.001**
Psychotic disorders	1078 (3.97)	281 (2.26)	797 (5.41)	
Manic episodes and/or bipolar disorders	1751 (6.44)	375 (3.02)	1376 (9.33)	
Depressive symptoms and/or disorders	11 369 (41.84)	3037 (24.44)	8332 (56.51)	
Anxiety symptoms and/or disorders	6239 (22.96)	2017 (16.23)	4222 (28.64)	
Somatoform disorders	6758 (24.87)	2729 (21.96)	4029 (27.33)	
**Psychiatric hospitalization, *N*** (**%)**^a^	2364 (8.70)	365 (2.94)	1030 (6.99)	**<0.001**

s.d., standard deviation; KRW, Korean Won; N/A, not applicable; CAD, coronary artery disease.

a Numbers and proportions of the participants who had at least one corresponding diagnostic record at the end of follow-up are provided.

### Association between antidepressant exposure and CVD

The mean of the log-transformed value of the stabilized IPT weights for the main analysis was −0.030 (s.d. 0.340) at baseline and −0.377 (s.d. 0.472) at the fourth time interval. The mean of the log-transformed value of the non-stabilized IPT weights for the main analysis was 7.444 (s.d. 0.820) at baseline and 8.572 (s.d. 0.759) at the fourth time interval (online Supplementary Material 4). The mean of the log-transformed value of the stabilized IPT weights for the dose–response analysis was −0.007 (s.d. 0.606) at baseline and −0.006 (s.d. 0.628) at the fourth time interval. The mean of the log-transformed value of the non-stabilized IPT weights for the dose–response analysis was 1.189 (s.d. 0.680) at baseline and 1.247 (s.d. 0.702) at the fourth time interval (online Supplementary Material 5).

**Figure 2.**
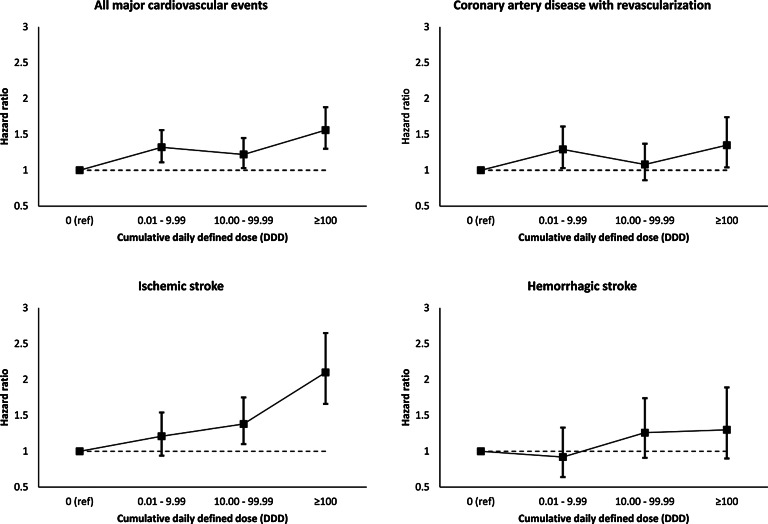
Dose response relationship between cumulative daily defined dose of antidepressant medications and cardiovascular disease

Antidepressant exposure was associated with incident MACE (hazard ratio **[HR] 1.34, 95% CI 1.18–1.53, e-value = 2.01**). When disaggregated by component CVD outcomes, there was an increased risk of coronary artery disease with revascularization (**HR 1.45, 95% CI 1.22–1.73, e-value = 2.26**) and ischaemic stroke (**HR 1.32, 95% CI 1.10–1.58, e-value = 1.97**) but no statistically significant increased risk of haemorrhagic stroke (HR 1.08, 95% CI 0.83–1.40) (Table 2). When disaggregated by antidepressant medication type, SSRIs (**HR 1.24, 95% CI 1.06–1.56, e-value = 1.79**) and TCAs (**HR 1.33, 95% CI 1.13–1.56, e-value = 1.99**) were both associated with an increased risk of MACE, while SNRIs (HR 1.08, 95% CI 0.84–1.39) showed no statistically significant association with MACE. The marginal structural model estimates were generally larger than those derived from the conventional time-fixed and time-varying survival analysis models, suggesting that time-varying confounding might bias the estimated treatment effects towards the null (Table 2 and Fig. 2). The e-values estimated from the analyses indicate that an unmeasured confounder would need to have a strength of association with both exposure and outcome equivalent to a HR of 1.79–2.26 in order to fully explain away the estimated exposure-outcome association (VanderWeele & Ding, 2017). Progressively truncating the stabilized IPT weights did not yield substantive alterations in the estimated HR, suggesting that the magnitude of possible bias due to violations of positivity was minimal (online Supplementary Material 6). Exclusion of participants with specific psychiatric comorbidities modified the estimates slightly, but the differences were not statistically significant (online Supplementary Material 7).

**Table 2.** Effect of antidepressant medication use on cardiovascular disease among patients with posttraumatic stress disorder (*N* = 27 170)

	Incidence rate among exposed, per 1000 person-years	Incidence rate among unexposed, per 1000 person-years	Time-fixed model, HR (95% CI)	Time-varying model, HR (95% CI)	Marginal structural model, HR (95% CI)
All major cardiovascular events					
Any antidepressants	9.90	7.94	**1.25 (1.12–1.40)**	**1.15 (1.03–1.28)**	**1.34 (1.18–1.53)**
SSRI	9.69	8.73	**1.18 (1.10–1.26)**	1.08 (0.96–1.22)	**1.24 (1.08–1.44)**
SNRI	12.54	8.80	**1.20 (1.01–1.44)**	1.08 (0.88–1.33)	1.08 (0.84–1.39)
TCA	11.12	8.21	**1.22 (1.09–1.37)**	**1.22 (1.08–1.37)**	**1.33 (1.13–1.56)**
Coronary artery disease with revascularization					
Any antidepressants	5.11	3.88	**1.28 (1.10–1.48)**	**1.19 (1.03–1.37)**	**1.45 (1.22–1.73)**
SSRI	5.26	4.21	**1.26 (1.10–1.45)**	**1.43 (1.23–1.67)**	**1.28 (1.06–1.56)**
SNRI	6.84	4.41	**1.38 (1.11–1.71)**	1.24 (0.95–1.62)	1.10 (0.78–1.56)
TCA	5.76	4.09	**1.43 (1.24–1.65)**	**1.50 (1.28–1.75)**	**1.40 (1.13–1.73)**
Ischaemic stroke					
Any antidepressants	5.28	4.35	**1.18 (1.01–1.39)**	1.13 (0.97–1.33)	**1.32 (1.10–1.58)**
SSRI	4.89	4.88	0.94 (0.82–1.08)	0.92 (0.80–1.06)	1.09 (0.89–1.34)
SNRI	6.45	4.76	1.11 (0.90–1.39)	1.11 (0.89–1.39)	1.09 (0.80–1.52)
TCA	5.99	4.42	**1.33 (1.16–1.52)**	**1.21 (1.06–1.39)**	**1.29 (1.04–1.60)**
Haemorrhagic stroke					
Any antidepressants	2.42	2.75	1.04 (0.82–1.31)	1.06 (0.84–1.34)	1.08 (0.83–1.40)
SSRI	2.39	2.65	0.88 (0.73–1.07)	0.90 (0.73–1.08)	1.17 (0.87–1.56)
SNRI	3.31	2.50	1.18 (0.87–1.60)	1.17 (0.86–1.60)	1.12 (0.68–1.86)
TCA	2.43	2.62	1.03 (0.85–1.26)	0.99 (0.81–1.21)	1.03 (0.74–1.43)

HR, hazard ratio; CI, confidence interval; SSRI, selective serotonin reuptake inhibitor; SNRI, serotonin-norepinephrine reuptake inhibitor; TCA, tricyclic antidepressants.

All models were adjusted for age, sex, monthly insurance premium, body mass index, systolic blood pressure, diastolic blood pressure, fasting blood glucose, total serum cholesterol, psychotic symptoms and/or psychosis, manic episode and/or bipolar disorder, depressive symptoms/depressive disorders, anxiety-related disorders, and admission due to psychiatric symptoms. In the time-fixed model, baseline values of covariates were included. In the time-varying model, time-varying values of covariates were included. In marginal structural models, IPT weights by time interval were calculated using logistic regression, and observations were weighted with the estimated IPT weights by time interval.

The dose–response analysis showed that higher cumulative DDD of antidepressant medication exposure was positively associated with MACE (per 1 unit increase in log-transformed value of cumulative DDD, **HR 1.07, 95% CI 1.04–1.10**). The dose‒response relationship was estimated for SSRIs (**HR 1.06, 95% CI 1.02–1.09**) and TCAs (**HR 1.12, 95% CI 1.05–1.19**) but not for SNRIs (HR 0.98, 95% CI 0.93–1.04). For the component CVD outcomes, a dose‒response relationship was estimated for coronary artery disease with revascularization (**HR 1.04, 95% CI 1.00–1.08**) and ischaemic stroke (**HR 1.12, 95% CI 1.08–1.16**), but the estimate for haemorrhagic stroke (HR 1.04, 95% CI 0.99–1.11) was of borderline statistical significance (Table 3). The HR for MACE was the highest in the subgroup with the highest cumulative DDD (Fig. 2). The dose response was most prominent for ischaemic stroke but was also estimated for other outcomes. The results from polynomial splining for cumulative DDD of antidepressants also supported the existence of a dose‒response relationship between antidepressants and CVD (online Supplementary Material 8). Additional analyses showedthat antidepressant medication is positively associated with development of atherosclerosis (**HR = 1.31, 95% CI 1.15–1.49, e-value = 1.57**) and hypertension (**HR = 1.33, 95% CI 1.22–1.46, e-value = 1.74**), suggesting a potential pathophysiological mechanism explaining the observed adverse cardiovascular effects of antidepressant medication (online Supplementary Material 9).

**Table 3.** Dose response relationship between log-transformed cumulative daily defined dose (DDD) of antidepressant medications and cardiovascular disease

	Time-fixed model, HR (95% CI)	Time-varying model, HR (95% CI)	Marginal structural model, HR (95% CI)
All major cardiovascular events			
Any antidepressants	**1.05 (1.02–1.08)**	**1.04 (1.01–1.06)**	**1.07 (1.04–1.10)**
SSRI	**1.03 (1.00–1.05)**	1.02 (0.99–1.05)	**1.06 (1.02–1.09)**
SNRI	1.02 (0.98–1.05)	0.99 (0.95–1.04)	0.98 (0.93–1.04)
TCA	**1.09 (1.04–1.13)**	1.07 (1.03–1.12)	**1.12 (1.05–1.19)**
Coronary artery disease with revascularization			
Any antidepressants	**1.05 (1.02–1.09)**	**1.04 (1.01–1.07)**	**1.04 (1.00–1.08)**
SSRI	**1.03 (1.00–1.06)**	1.02 (0.99–1.06)	1.03 (0.98–1.07)
SNRI	1.04 (0.99–1.09)	1.01 (0.95–1.07)	0.98 (0.91–1.07)
TCA	**1.09 (1.04–1.15)**	**1.08 (1.02–1.14)**	**1.15 (1.06–1.25)**
Ischaemic stroke			
Any antidepressants	**1.05 (1.01–1.08)**	**1.04 (1.01–1.08)**	**1.12 (1.08–1.16)**
SSRI	1.01 (0.98–1.05)	1.01 (0.97–1.04)	**1.10 (1.06–1.15)**
SNRI	1.00 (0.95–1.05)	1.01 (0.95–1.07)	0.97 (0.90–1.05)
TCA	**1.12 (1.06–1.18)**	**1.10 (1.04 −1.16)**	**1.12 (1.03–1.21)**
Haemorrhagic stroke			
Any antidepressants	0.95 (0.90–1.00)	0.98 (0.93–1.03)	1.04 (0.99–1.11)
SSRI	0.97 (0.92–1.02)	1.00 (0.95–1.05)	1.04 (0.98–1.10)
SNRI	0.95 (0.87–1.03)	0.92 (0.83–1.02)	0.99 (0.88–1.11)
TCA	0.98 (0.89–1.08)	1.00 (0.91–1.10)	1.04 (0.91–1.20)

HR, hazard ratio; CI, confidence interval; SSRI, selective serotonin reuptake inhibitor; SNRI, serotonin-norepinephrine reuptake inhibitor; TCA, tricyclic antidepressants.

All models were adjusted for age, sex, monthly insurance premium, body mass index, systolic blood pressure, diastolic blood pressure, fasting blood glucose, total serum cholesterol, psychotic symptoms and/or psychosis, manic episode and/or bipolar disorder, depressive symptoms/depressive disorders, anxiety-related disorders, and admission due to psychiatric symptoms. In the time-fixed model, baseline values of covariates were included. In the time-varying model, time-varying values of covariates were included. In marginal structural models, IPT weights by time interval were calculated using logistic regression, and observations were weighted with the estimated IPT weights by time interval. Bold indicates *p*-value < 0.05.

## Discussion

This marginal structural model analysis of data from a nationally representative cohort of adults accessing the South Korean health care system showed that exposure to antidepressant medications among people with PTSD was associated with an increased risk of MACE. The increase in risk appeared to be driven primarily by SSRIs and TCAs and increased in a dose-dependent fashion. Confounding by an unmeasured variable would need to be strong in order to explain away the observed effects.

There is some controversy in the literature about whether antidepressant use increases or decreases the risk of CVD. Antidepressants can improve psychiatric symptoms and consequently decrease the inflammatory response and risk of CVD, (Edmondson & Cohen, 2013; Mellon, Gautam, Hammamieh, Jett, & Wolkowitz, 2018) but they can also increase CVD risk due to potential cardiotoxic side effects, which may lead to an increased risk of ischaemic heart disease (Cohen et al., 2000; Grace et al., 2018; Jang et al., 2020) and ischaemic stroke (Gaffey et al., 2021; Glymour et al., 2019; Smoller et al., 2009). For instance, TCAs are known to have a potential association with electrocardiographic abnormalities, such as elongation of the rate-corrected QT interval and widening of the QRS complex, (Littmann, 2021) that can cause myocardial infarction and stroke (Jang et al., 2020). Some reports of SSRIs and SNRIs also suggest that low serotonergic activity is associated with atherosclerotic changes, (Muldoon et al., 2007; Sauer, Berlin, & Kimmel, 2003) but these effects might differ by characteristics of the population. Drawing causal conclusions about the potential cardiovascular effects of antidepressant medication exposure is further complicated due to confounding by indication: since people with severe psychiatric symptoms are more likely to be treated with antidepressant medications, it is difficult to distinguish between changes in psychiatric symptoms and the intended therapeutic effects of antidepressant treatment.

Compared with the existing literature on people with depressive disorders, there has been a comparative lack of evidence on cardiovascular risk in relation to antidepressant use for people with PTSD. Studies with appropriate control of time-varying confounding affected by prior treatment are essential for drawing causal conclusions; however, unlike with depressive disorders, (Glymour et al., 2019) such studies on people with PTSD are lacking. A major difference in psychopharmacologic treatment for PTSD and depressive disorder is that use of antidepressant medication is often intended as an adjunct to psychotherapeutic treatment: a network meta-analysis of treatment for PTSD suggested that psychotherapeutic interventions showed superior therapeutic effects compared to antidepressant medications alone (Merz, Schwarzer, & Gerger, 2019). In contrast, combination of pharmacologic and nonpharmacologic intervention is the most effective treatment modality in depressive disorder, and both modalities are believed to be equally important (Chen & Shan, 2019). Considering the differential treatment effect of antidepressant medications in depressive disorder *v.* PTSD, it could be expected that any CVD effects of antidepressant medication exposure are also likely to vary by disease entity.

While there has been some research on antidepressants and CVD among people with PTSD, (Gaffey et al., 2021; Scherrer et al., 2020) the time-dependent effects of antidepressant use and symptom severity have not been fully considered in these studies. Application of marginal structural modelling to data from a national cohort enabled us to adjust for time-varying confounding by psychiatric symptoms and comorbidities and to estimate effect sizes that are less likely to be affected by this confounding structure. Additionally, given the context of our study, treatment assignment is less likely to be subject to confounding by indication: antidepressant medication is a mainstay of treatment for depressive disorders in South Korea, (Kim et al., 2019) so people who do not receive antidepressant medication might have special characteristics; thus, findings about antidepressant medication exposure and CVD among people with depressive disorders would be more prone to bias. In contrast, since there is more clinical equipoise in regard to pharmacological treatment of PTSD, the use of antidepressant medication among people with PTSD might be less subject to confounding by indication (Merz et al., 2019).

Interpretation of our findings is subject to a number of limitations. First, as with other observational studies aimed at establishing a causal association, we assume that conditioning on the measured covariates was sufficient to achieve exchangeability between people who were and were not exposed to antidepressant medications during the study period, but the assumption of no unmeasured confounding is not empirically verifiable (Hernán, 2004). Nevertheless, considering that the e-value, which is defined as ‘the minimum strength of association that an unmeasured confounder would need to have with both exposure and outcome to fully explain away the association’, (VanderWeele & Ding, 2017) was 1.79 or larger for the estimates in our study, it is unlikely that unmeasured confounding wouldfully explain away the observed association between antidepressant medication exposure and CVD. Second, because the Korean NHID is an administrative database, we did nto have access to important clinical data, such as biological markers (e.g. markers of inflammation and autonomic nervous system functioning) and symptom severity. Thus, our analysis is mostly agnostic about the potential biological mechanisms underlying the observed antidepressant-CVD association, as well as effects of symptom severity on cardiovascular outcome. Third, there could have been misclassification in the outcome. Previous validation studies of CVD outcomes based on diagnostic codes in the NHID have been conducted, (Park & Choi, 2016) but it is possible that nonfatal CVD events are subject to undercounting. Fourth, we assumed positivity, which is a reasonable assumption given that there were no factors in the setting of our data that would have been completely deterministic of antidepressant medication treatment (or nontreatment). Although it is not possible to directly check if positivity assumption is applicable, it is able to estimate if the bias introduced by breaking the assumption significantly alters the estimand. We tested for possible bias from violations of positivity by progressively truncating the stabilized IPT weights and re-estimating the parameters of the marginal structural models, but doing so did not alter the direction or the magnitude of the estimated associations. We also ruled out the possibility of random zeroes by identifying treated and untreated study participants at each level of psychiatric symptomatology and comorbidity in the data. Fifth, since our study is based on insurance claim data rather than clinical data, medication compliance is likely to be overestimated. The magnitude of bias is expected to be stronger for patients with more severe symptoms, (Scherrer et al., 2020) but we were unable to estimate the magnitude of this bias given the limitations of the database. Finally, our findings might not be generalizable to other populations. According to the Korean NHIS, approximately 98% of Korean residents covered by Korean National Health Insurance are of Korean nationality (Kim & Kim, 2019), and more than 99% of Korean nationals are of Korean ethnicity (Ministry of the Interior and Safety, 2019). Currently, there are insufficient existing studies on ethnic variation in cardiovascular outcomes associated with antidepressant medication use, with the bulk of prior studies derived from study populations that are predominantly Caucasian (Glymour et al., 2019; Grace et al., 2018; Monster, Johnsen, Olsen, McLaughlin, & Sorensen, 2004; Rubin et al., 2010). Previous studies have suggested ethnic differences in pharmacogenetics, which can result in variations in antidepressant response (Kato & Serreti, 2010; Lesser et al., 2010). Since virtually all participants covered by the Korean NHIS are of Korean nationality and ethnicity, the findings from this study may not generalize to other ethnic groups.

## Conclusions

Our study provides evidence of a possible link between antidepressant medication use and cardiovascular risk among people with PTSD. Our analysiscontrols for time-dependent confounding due to psychiatric symptoms and comorbidity affected by prior treatment and can be generalized to the Korean population. For people with PTSD who are at high risk of CVD, a thorough CVD risk assessment might be considered prior to initiating treatment with antidepressant medications.

## Supporting information

Kim et al. supplementary material Kim et al. supplementary material

## Data Availability

Due to the data sharing policy of the Korean National Health Insurance Service, registration and authorization are required to gain access to the data.

## Supplementary material

The supplementary material for this article can be found at https://doi.org/10.1017/S0033291723001873.

## Additional information

A prelimlinary version of this study was presented at the Annual Congress of the Association of Cardiovascular Nursing and Allied Professions-EuroHeartCare 2022 Conference and was published in an online supplement of the *European Journal of Cardiovascular Nursing*, Volume 21 (Kim, Kang, & Jung, 2022).
