# Supplementary material for: A marginal structural model to estimate the effect of antidepressant medication treatment on major cardiovascular events among people with post-traumatic stress disorder
Source: Psychol Med. 2023 Jul 24;53(16):7837–46. doi: 10.1017/S0033291723001873 (PMC10755244; doi:10.1017/S0033291723001873)
Supplement: Kim et al. supplementary material [file S0033291723001873sup001.docx]

**Supplemental Material 1. Flow chart of participant inclusion and exclusion.**

Patients diagnosed with PTSD in years 2004-2018

(N = 109,235)

Aged 18 or younger at PTSD diagnosis (N = 35,067)

Missing or erroneous eligibility data (N = 5)

Never received medical checkup from NHIS (N = 15,041)

Prior diagnosis of cardiovascular disease (N = 3,107)

Adult patients with PTSD who received medical checkup
(N = 59,122)

Patients included in final analysis (N = 27,170)

Received antidepressants before diagnosis of PTSD (N = 28,845)

**Supplemental Material 2. List of pharmaceutical codes of ingredients for antidepressants**

| **Category** | **Pharmaceutical code of ingredients** | **Pharmaceutical contents** |
| --- | --- | --- |
| **SSRI** | 161501ACH | fluoxetine hydrochloride (as fluoxetine 10mg) |
|  | 161501ATB | fluoxetine hydrochloride (as fluoxetine 10mg) |
|  | 161502ACH | fluoxetine hydrochloride (as fluoxetine 20mg) |
|  | 161502ATB | fluoxetine hydrochloride (as fluoxetine 20mg) |
|  | 161502ATD | fluoxetine hydrochloride (as fluoxetine 20mg) |
|  | 162501ATB | fluvoxamine maleate 50mg |
|  | 162502ATB | fluvoxamine maleate 0.1g |
|  | 209301ATB | paroxetine hydrochloride (as paroxetine 10mg) |
|  | 209302ATB | paroxetine hydrochloride (as paroxetine 20mg) |
|  | 209304ATR | paroxetine hydrochloride (as paroxetine 12.5mg) |
|  | 209305ATR | paroxetine hydrochloride (as paroxetine 25mg) |
|  | 227001ATB | sertraline hydrochloride (as sertraline 50mg) |
|  | 227002ATB | sertraline hydrochloride (as sertraline 0.1g) |
|  | 227003ATB | sertraline hydrochloride (as sertraline 25mg) |
|  | 229601ATB | sodium tianeptine 12.5mg |
|  | 428301ATB | citalopram hydrobromide (as citalopram 20mg) |
|  | 474801ATB | escitalopram oxalate (as escitalopram 5mg) |
|  | 474802ATB | escitalopram oxalate (as escitalopram 10mg) |
|  | 474803ATB | escitalopram oxalate (as escitalopram 20mg) |
|  | 474804ATB | escitalopram oxalate (as escitalopram 15mg) |
| **SNRI** | 247502ACR | venlafaxine hydrochloride (as venlafaxine 75mg) |
|  | 247504ACR | venlafaxine hydrochloride (as venlafaxine 37.5mg) |
|  | 355801ACH | milnacipran hydrochloride 25mg |
|  | 355802ACH | milnacipran hydrochloride 50mg |
|  | 355803ACH | milnacipran hydrochloride 12.5mg |
|  | 247502ACR | venlafaxine hydrochloride (as venlafaxine 75mg) |
|  | 247504ACR | venlafaxine hydrochloride (as venlafaxine 37.5mg) |
|  | 355801ACH | milnacipran hydrochloride 25mg |
|  | 355802ACH | milnacipran hydrochloride 50mg |
|  | 495501ACE | duloxetine hydrochloride (as duloxetine 30mg) |
|  | 495501ATE | duloxetine hydrochloride (as duloxetine 30mg) |
|  | 495502ACE | duloxetine hydrochloride (as duloxetine 60mg) |
|  | 495502ATE | duloxetine hydrochloride (as duloxetine 60mg) |
|  | 626401ATR | desvenlafaxine succinate monohydrate (as desvenlafaxine 50mg) |
|  | 626402ATR | desvenlafaxine succinate monohydrate (as desvenlafaxine 100mg) |
| **TCA** | 107501ATB | amitriptyline hydrochloride 10mg |
|  | 107502ATB | amitriptyline hydrochloride 25mg |
|  | 107504ATB | amitriptyline hydrochloride 5mg |
|  | 108002ATB | amoxapine 50mg |
|  | 136301ACH | clomipramine hydrochloride 10mg |
|  | 136302ACH | clomipramine hydrochloride 25mg |
|  | 173701ATB | imipramine hydrochloride 25mg |
|  | 203401ATB | nortriptyline hydrochloride (as nortriptyline 10mg) |
|  | 203402ATB | nortriptyline hydrochloride (as nortriptyline 25mg) |

SSRI, selective serotonin reuptake inhibitor; SNRI, serotonin-norepinephrine reuptake inhibitor; TCA, tricyclic antidepressants.

**Supplemental Material 3. Statistical methods for calculating inverse probability weights (IPTWs) for main analysis and dose-response analysis.**

To calculate IP weights, we used logistic regression to estimate the associations between covariates and the probability of antidepressant use in 3-month time intervals. The stabilized inverse probability (IP) of the exposure weight was estimated as follows:

${AW}_{i} =\frac{\prod_{k=0}^{K_{i}} pr\left( A_{k} =a_{ki} \right|\bar{A_{k-1}}=\bar{a_{\left( k-1 \right)i}})}{\prod_{k=0}^{K_{i}} pr\left( A_{k} =a_{ki} \right|\bar{A_{k-1}}=\bar{a_{\left( k-1 \right)i}}, \bar{L_{k}}=\bar{l_{ki}})}$ (1)

(${AW}_{i}$: Stabilized IP weight for antidepressant exposure; A_k_: Antidepressant exposure at time interval $I_{k}=[t_{k}, t_{k+1}]$; $\bar{A_{k-1}}$: Column vector of antidepressant exposure during time interval $I_{1}\sim I_{k-1}$; L_k_: Covariates at time interval $I_{k}=[t_{k-1}, t_{k}]$; $\bar{L_{k-1}}$: Column vector of covariates during time interval $I_{0}\sim I_{k-1}$; $K_{i}$: Maximum number of time intervals for individual I; $t_{k}$: start time of time interval $I_{k}$; $t_{k+1}$: end time of time interval $I_{k}$)

The stabilized IP of censoring weight was estimated similarly: (Hernán *et al.*, 2000, Robins *et al.*, 2000)

${CW}_{i} =\frac{\prod_{k=0}^{K_{i}} pr\left( Y_{k} =y_{ki} \right|\bar{Y_{k-1}}=\bar{y_{\left( k-1 \right)i}}, \bar{A_{k}}=\bar{a_{ki}} )}{\prod_{k=0}^{K_{i}} pr\left( Y_{k} =y_{ki} \right|\bar{Y_{k-1}}=\bar{y_{\left( k-1 \right)i}}, \bar{A_{k}}=\bar{a_{ki}}, \bar{L_{k}}=\bar{l_{ki}})}$ (2)

(${CW}_{i}$: Stabilized IP weight for censoring; Y_k_: censoring at time interval $I_{k}=[t_{k}, t_{k+1}]$; $\bar{Y_{k-1}}$: Column vector of censoring during time interval $I_{1}\sim I_{k-1}$)

Exposure and covariate status were updated every 3 months since the index date. All models included time from enrolment modelled as a restricted cubic spline with knots at 5p, 25p, 50p, 75p, and 95p. (Glymour *et al.*, 2019a) The final IP weight was defined as the product of ${AW}_{i}$and ${CW}_{i}$. The final IP weights were applied to a discrete-time survival analysis model to estimate the hazard ratio (HR) and 95% confidence interval (CI) associated with ever being exposed to antidepressant medication treatment on the composite MACE outcome. We also conducted disaggregated analyses to estimate the effects of treatment with SSRIs, SNRIs, and TCAs specifically. In these analyses by antidepressant classes, the comparator group included all participants not exposed to the antidepressant class of interest. For instance, when estimating effects of SSRI on MACE, comparator group includes all participants not exposed to SSRI, including those who were exposed to SNRI and/or TCA.

For comparison, conventional time-fixed and time-varying Cox regression models were also fitted. In time-fixed Cox regression, baseline values of covariates were adjusted. In time-varying Cox regression, time-varying values of covariates obtained from follow-up data were adjusted. We estimated HRs and 95% CIs for each disease entity, except for cardiovascular mortality due to low statistical power and inability to obtain stable estimates from the database.

We log-transformed the cumulative DDD and estimated the HR associated with cumulative DDD on the composite MACE outcome. To test for the presence of a dose‒response relationship between cumulative DDD and risk for cardiovascular disease, participants were categorized into 4 subgroups in accordance of cumulative DDD of antidepressant medications: 0 (reference value), 0.01 – 9.99, 10.00 – 99.99, and 100 or higher. Multinomial logistic regression was conducted to estimate probabilities of participants being allocated to either of 4 subgroups, and IP weight for each time interval was estimated.

${AW}_{i}' =\frac{\prod_{k=0}^{K_{i}} pr\left( A_{k}' =a_{ki}' \right|\bar{A_{k-1}}=\bar{a_{\left( k-1 \right)i}}')}{\prod_{k=0}^{K_{i}} pr\left( A_{k}' =a_{ki} \right|\bar{A_{k-1}}'=\bar{a_{\left( k-1 \right)i}}', \bar{L_{k}}=\bar{l_{ki}})}$ (3)

(${AW}_{i}'$: Stabilized IP weight for cumulative DDD of antidepressant medication; $A_{k}'$: cumulative DDD of antidepressant medication at time interval $I_{k}=[t_{k}, t_{k+1}]$; $\bar{A_{k-1}}'$: Column vector of cumulative DDD of antidepressant medication during time interval $I_{1}\sim I_{k-1}$)

IP weight for dose-response analysis was calculated as the product of ${AW}_{i}'$and ${CW}_{i}$instead of the product of ${AW}_{i}$and ${CW}_{i}$.

**Supplemental Material 4. Distribution of inverse probability weights for main analysis at each time interval.**

**A) Stabilized weight B) Non-stabilized weight**


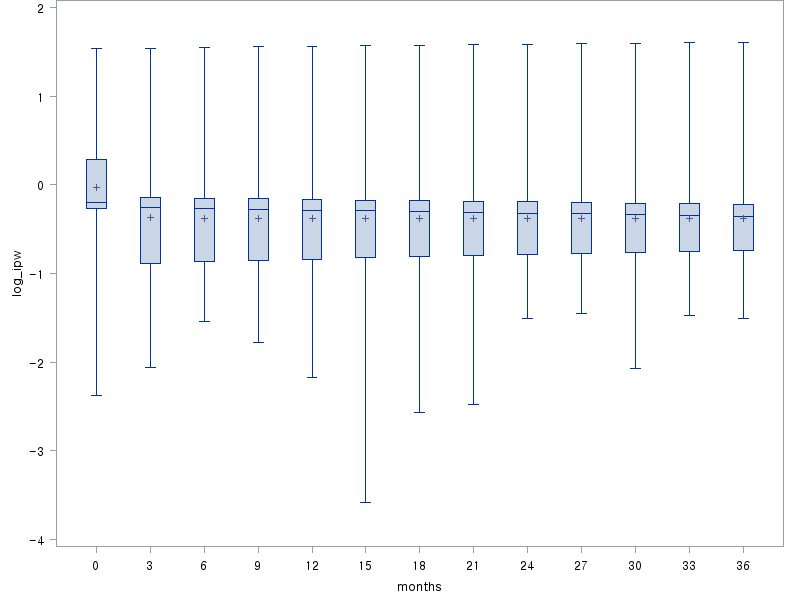

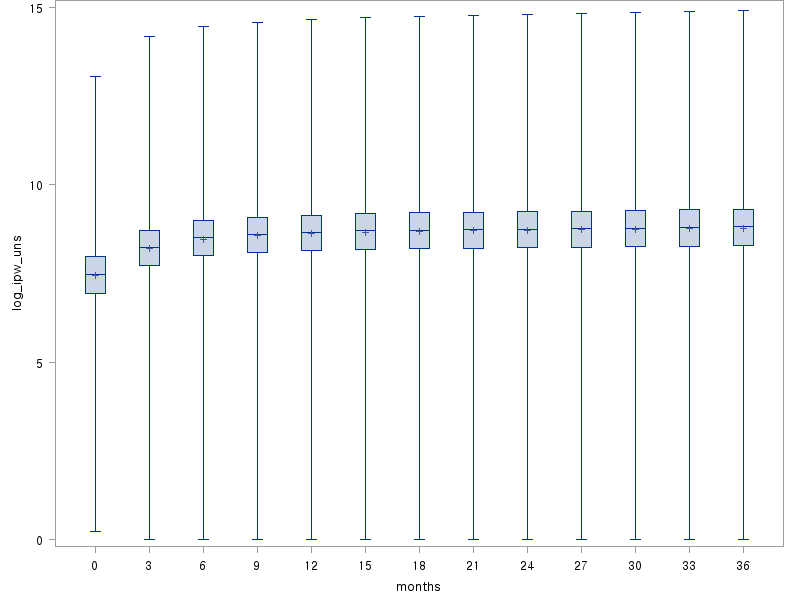


**Supplemental Material 5. Distribution of inverse probability weights for dose-response analysis at each time interval.**

**A) Stabilized weight B) Non-stabilized weight**


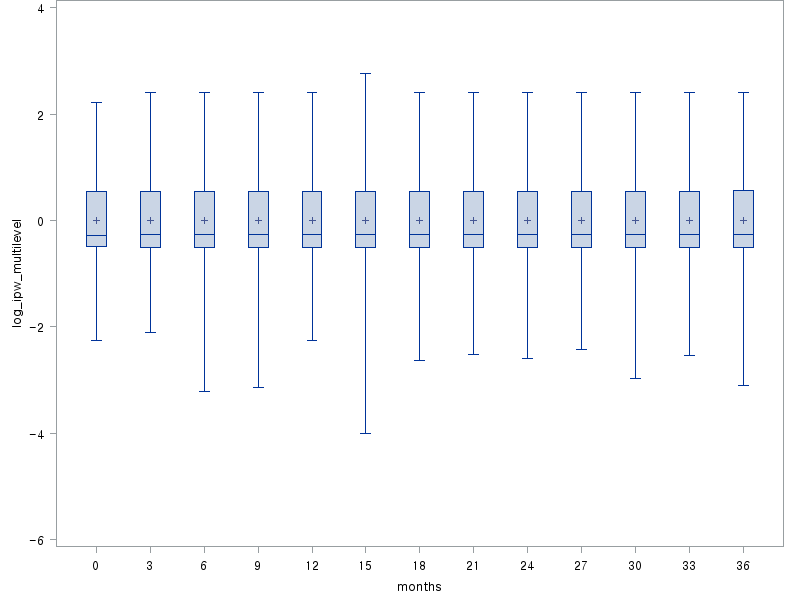

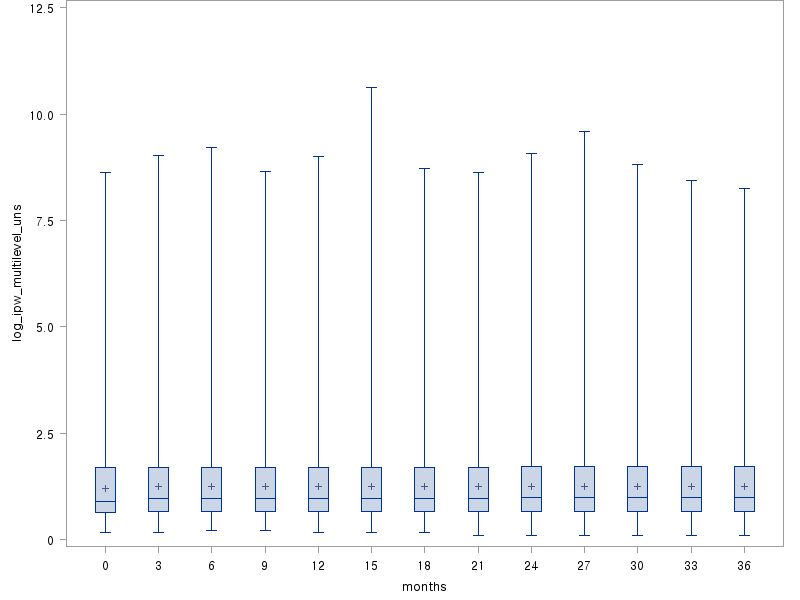


**Supplemental Material 6. Results from sensitivity analyses with different weight truncation.**

| **Weight truncation** | **No truncation** | **1% - 99%** | **5% - 95%** | **10% - 90%** |
| --- | --- | --- | --- | --- |
| **All major cardiovascular events** | | | | |
| Any antidepressants | **1.34 (1.18 - 1.53)** | **1.37 (1.20 – 1.55)** | **1.33 (1.16 – 1.52)** | **1.34 (1.16 – 1.55)** |
| SSRI | **1.24 (1.08 - 1.44)** | **1.23 (1.06 – 1.42)** | **1.20 (1.03 – 1.39)** | **1.20 (1.02 – 1.41)** |
| SNRI | 1.08 (0.84 - 1.39) | 1.11 (0.86 – 1.43) | 1.13 (0.87 – 1.47) | 1.16 (0.87 – 1.54) |
| TCA | **1.33 (1.13 - 1.56)** | **1.35 (1.15 – 1.58)** | **1.31 (1.11 – 1.54)** | **1.34 (1.13 – 1.59)** |
| **Coronary artery disease with revascularization** | | | | |
| Any antidepressants | **1.45 (1.22 - 1.73)** | **1.49 (1.25 – 1.77)** | **1.41 (1.18 – 1.69)** | **1.39 (1.15 – 1.69)** |
| SSRI | **1.28 (1.06 - 1.56)** | **1.38 (1.14 – 1.68)** | **1.29 (1.05 – 1.58)** | **1.28 (1.03 – 1.58)** |
| SNRI | 1.10 (0.78 - 1.56) | 1.03 (0.72 – 1.46) | 1.09 (0.75 – 1.58) | 1.13 (0.76 – 1.69) |
| TCA | **1.40 (1.13 - 1.73)** | **1.31 (1.06 – 1.62)** | **1.31 (1.05 – 1.64)** | **1.30 (1.03 – 1.64)** |
| **Ischaemic stroke** |  |  |  |  |
| Any antidepressants | **1.32 (1.10 – 1.58)** | **1.31 (1.09 – 1.53)** | **1.23 (1.01 – 1.45)** | **1.26 (1.03 – 1.52)** |
| SSRI | 1.09 (0.89 - 1.34) | 1.09 (0.89 – 1.34) | 1.01 (0.76 – 1.57) | 1.02 (0.82 – 1.29) |
| SNRI | 1.09 (0.80 - 1.52) | 1.08 (0.77 – 1.52) | 1.10 (0.76 – 1.57) | 1.14 (0.77 – 1.68) |
| TCA | **1.29 (1.04 - 1.60)** | **1.28 (1.03 – 1.58)** | **1.22 (1.00 – 1.57)** | **1.27 (1.01 – 1.62)** |
| **Haemorrhagic stroke** |  |  |  |  |
| Any antidepressants | 1.08 (0.83 - 1.40) | 1.09 (0.84 – 1.42) | 1.01 (0.77 – 1.33) | 1.10 (0.82 – 1.47) |
| SSRI | 1.17 (0.87 - 1.56) | 1.18 (0.87 – 1.58) | 1.08 (0.80 – 1.48) | 1.20 (0.86 – 1.66) |
| SNRI | 1.12 (0.68 - 1.86) | 1.11 (0.66 – 1.85) | 1.26 (0.75 – 2.13) | 1.33 (0.77 – 2.32) |
| TCA | 1.03 (0.74 - 1.43) | 1.03 (0.74 – 1.44) | 0.99 (0.70 – 1.40) | 0.95 (0.66 – 1.37) |

**Supplemental Material 7. Sensitivity analysis results excluding participants with psychiatric comorbidities.**

| Excluded psychiatric comorbidity  (Number of participants after exclusion) | Full cohort  (N = 27,170) | Psychotic disorders (N = 26,092) | Manic episodes and bipolar disorders (N = 25,419) | Anxiety symptoms and disorders (N = 20,931) | Somatoform disorders (N = 20,412) |
| --- | --- | --- | --- | --- | --- |
| **All major cardiovascular events** | | | | | |
| Any antidepressants | **1.34 (1.18 – 1.53)** | **1.34 (1.18 - 1.53)** | **1.34 (1.18 - 1.52)** | **1.35 (1.17 - 1.55)** | **1.35 (1.17 - 1.55)** |
| SSRI | **1.24 (1.08 – 1.44)** | **1.20 (1.04 - 1.37)** | **1.21 (1.05 - 1.39)** | **1.24 (1.06 - 1.45)** | 1.15 (0.98 - 1.35) |
| SNRI | 1.08 (0.84 – 1.39) | 1.16 (0.91 - 1.48) | 1.09 (0.84 - 1.41) | 1.15 (0.86 - 1.53) | 1.01 (0.74 - 1.37) |
| TCA | **1.33 (1.13 – 1.56)** | **1.34 (1.15 - 1.55)** | **1.33 (1.14 - 1.54)** | **1.30 (1.09 - 1.54)** | **1.40 (1.17 - 1.68)** |
| **Coronary artery disease with revascularization** | | | | | |
| Any antidepressants | **1.45 (1.22 – 1.73)** | **1.44 (1.18 - 1.76)** | **1.44 (1.19 - 1.75)** | **1.44 (1.18 - 1.75)** | **1.30 (1.04 - 1.54)** |
| SSRI | **1.28 (1.06 – 1.56)** | **1.28 (1.04 - 1.57)** | **1.32 (1.08 - 1.63)** | **1.29 (1.02 - 1.62)** | 1.16 (0.91 - 1.49) |
| SNRI | 1.10 (0.78 – 1.56) | 1.15 (0.79 - 1.16) | 1.02 (0.68 - 1.52) | 1.27 (0.83 - 1.94) | 1.17 (0.75 - 1.82) |
| TCA | **1.40 (1.13 – 1.73)** | **1.40 (1.10 - 1.79)** | **1.41 (1.13 - 1.77)** | **1.38 (1.07 - 1.80)** | **1.48 (1.15 - 1.89)** |
| **Ischaemic stroke** | | | | | |
| Any antidepressants | **1.32 (1.10 - 1.58)** | **1.31 (1.09 - 1.58)** | **1.31 (1.08 - 1.58)** | **1.29 (1.05 - 1.59)** | **1.34 (1.08 - 1.66)** |
| SSRI | 1.09 (0.89 - 1.34) | 1.10 (0.89 - 1.37) | 1.06 (0.86 - 1.32) | 1.13 (0.89 - 1.44) | 1.14 (0.78 - 1.30) |
| SNRI | 1.09 (0.80 - 1.52) | 1.16 (0.82 - 1.62) | 1.15 (0.81 - 1.62) | 0.86 (0.55 - 1.34) | 0.65 (0.39 - 1.09) |
| TCA | **1.29 (1.04 - 1.60)** | **1.25 (1.00 - 1.56)** | **1.32 (1.06 - 1.64)** | **1.25 (1.00 - 1.56)** | **1.49 (1.16 - 1.93)** |
| **Haemorrhagic stroke** | | | | | |
| Any antidepressants | 1.08 (0.83 – 1.40) | 1.12 (0.85 - 1.47) | 1.12 (0.85 - 1.47) | 1.08 (0.81 - 1.47) | 1.28 (0.93 - 1.77) |
| SSRI | 1.17 (0.87 – 1.56) | 1.25 (0.92 - 1.70) | 1.20 (0.88 - 1.63) | 1.11 (0.79 - 1.56) | 1.38 (0.97 - 1.97) |
| SNRI | 1.12 (0.68 – 1.86) | 1.22 (0.72 - 2.08) | 1.27 (0.74 - 2.16) | 1.23 (0.66 - 2.29) | 1.16 (0.62 - 2.17) |
| TCA | 1.03 (0.74 – 1.43) | 1.04 (0.74 - 1.47) | 1.07 (0.76 - 1.51) | 1.14 (0.78 - 1.66) | 1.14 (0.76 - 1.72) |

HR, hazard ratio; CI, confidence interval; SSRI, selective serotonin reuptake inhibitor; SNRI, serotonin-norepinephrine reuptake inhibitor; TCA, tricyclic antidepressants.

All estimates were derived from marginal structural model. Inverse probability weights by time interval were calculated by logistic regression model and individuals were weighted with estimated inverse probability weights by time interval.

**Supplemental Material 8. Polynomial splining for dose response estimation between cumulative daily defined dose of antidepressants and major cardiovascular events.**

**(A) All major cardiovascular event (B) Coronary artery disease with revascularization**

**
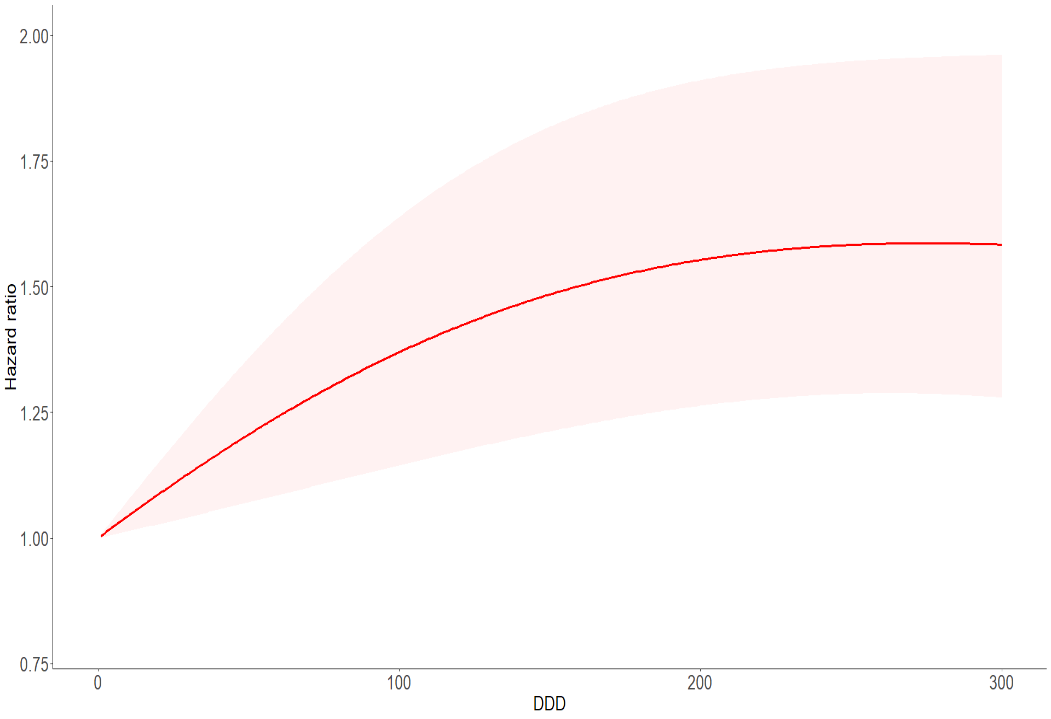

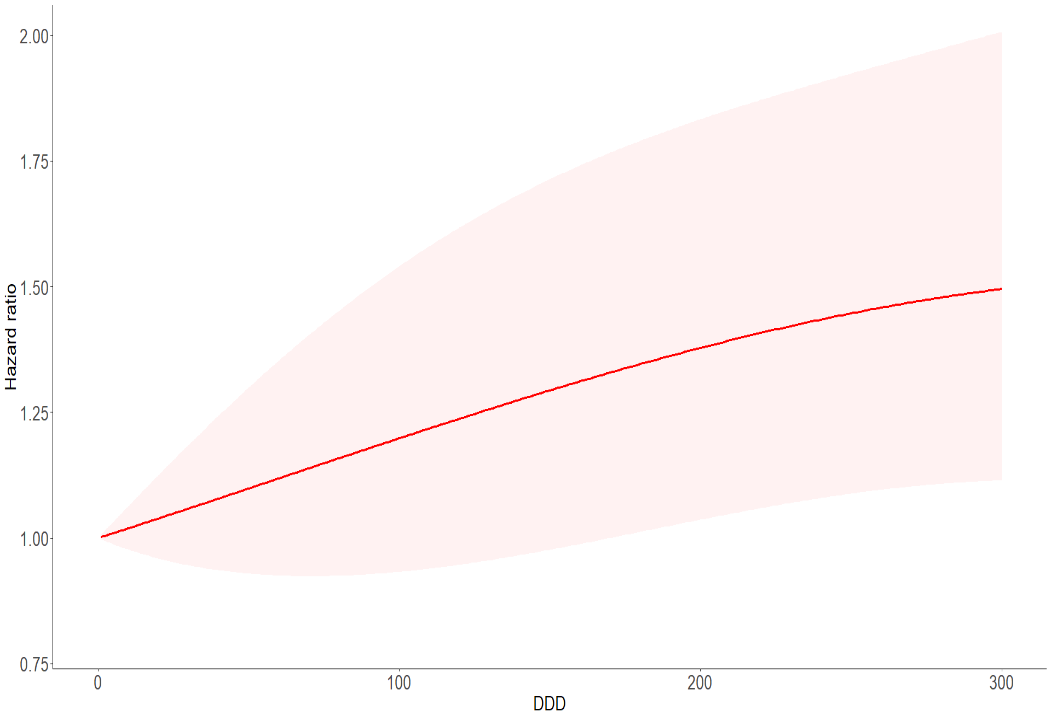
**

**(C) Ischaemic stroke (D) Haemorrhagic stroke**

**
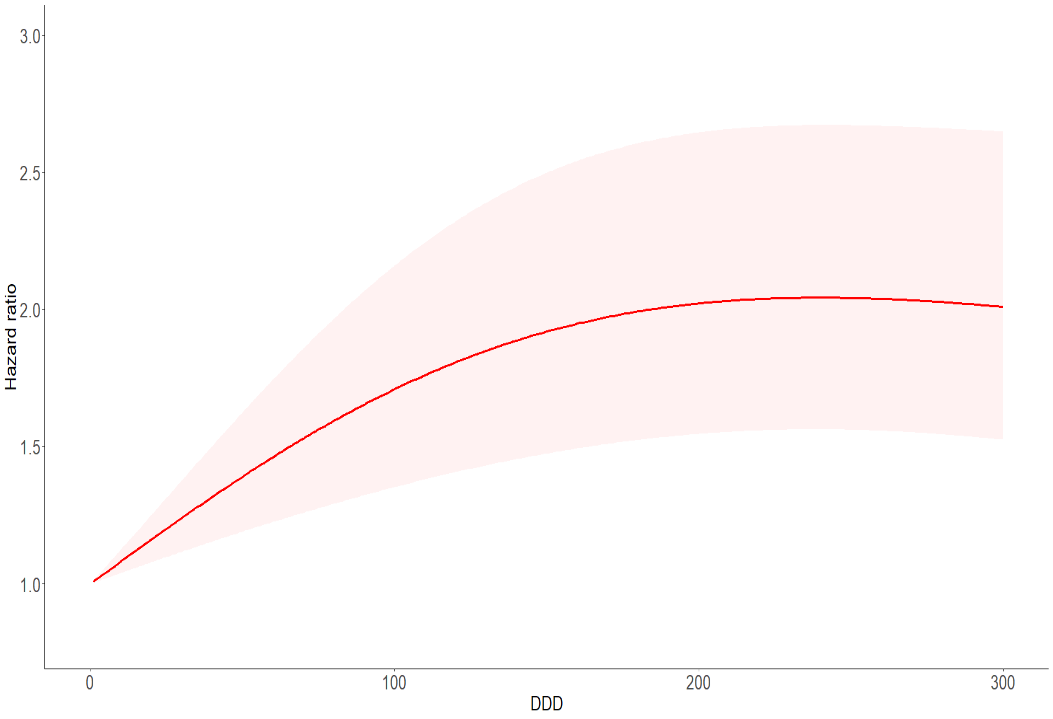

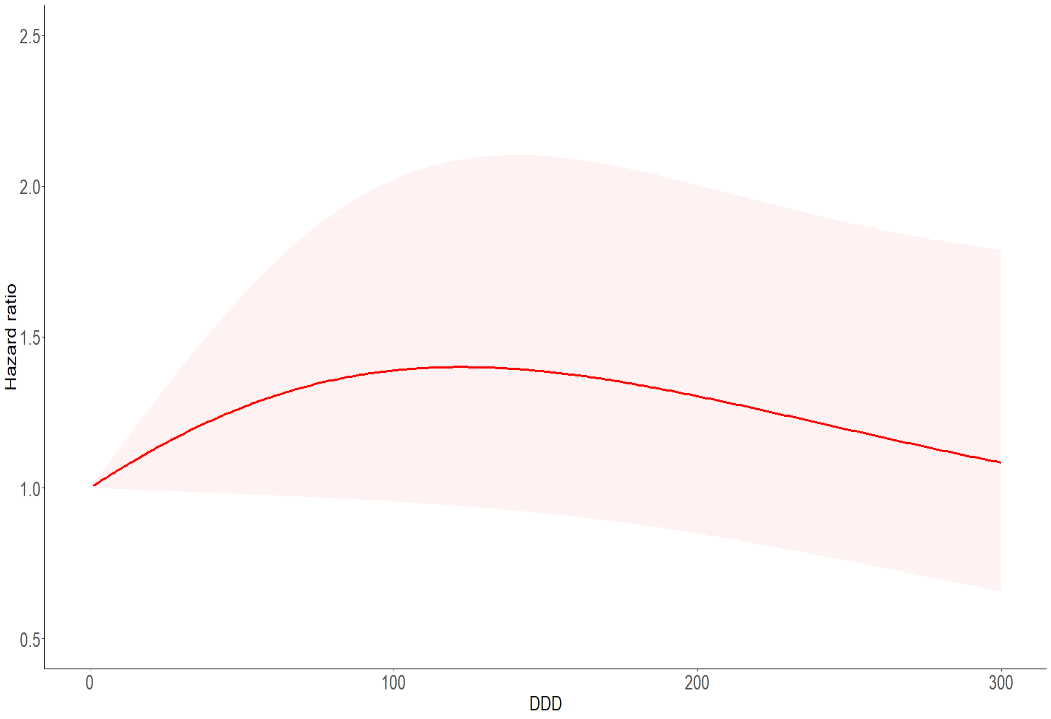
**

**Supplemental Material 9. Association between antidepressant medication and CVD risk factors (incident atherosclerosis, hypertension, and diabetes mellitus).**

|  | **Atherosclerosis, HR (95% CI)** | **Hypertension, HR (95% CI)** | **Diabetes mellitus, HR (95% CI)** |
| --- | --- | --- | --- |
| Any antidepressant | **1.31 (1.15 - 1.49)** | **1.33 (1.22 - 1.46)** | 1.07 (0.76 - 1.50) |
| SSRI | 1.11 (0.95 - 1.28) | 0.99 (0.89 - 1.10) | 1.19 (0.81 - 1.75) |
| SNRI | 1.08 (0.82 - 1.42) | 1.24 (1.01 - 1.51) | 0.60 (0.22 - 1.65) |
| TCA | **1.41 (1.20 - 1.65)** | **1.55 (1.39 - 1.74)** | 0.94 (0.58 - 1.51) |

HR, hazard ratio; CI, confidence interval; SSRI, selective serotonin reuptake inhibitor; SNRI, serotonin-norepinephrine reuptake inhibitor; TCA, tricyclic antidepressants.

All estimates were derived from marginal structural model. Inverse probability weights by time interval were calculated by logistic regression model and individuals were weighted with estimated inverse probability weights by time interval.

**Supplemental Material 10. Comparison of characteristics of participants included and excluded from final analysis.**

|  | **Total (N = 27,170)** | **Excluded (N = 35,576)** | **p-value** |
| --- | --- | --- | --- |
| **Age at index date, mean (SD)** | 39.84 (12.53) | 41.86 (12.97) | **<0.001** |
| **Male, N (%)** | 11,023 (40.57) | 12,563 (35.31) | **<0.001** |
| **Monthly insurance premium, KRW, N (%)** |  |  | **<0.001** |
| 0 (Medicaid recipients) | 910 (3.35) | 2,050 (5.76) |  |
| ≤20p (1-15,300) | 4,721 (17.38) | 3,388 (9.52) |  |
| 20 - 40p (15,301 - 27,300) | 5,787 (21.30) | 5,320 (14.95) |  |
| 40 - 60p (27,301 - 41,000) | 5,357 (19.72) | 5,369 (15.09) |  |
| 60 - 80p (41,001 - 59,300) | 5,394 (19.85) | 5,574 (15.67) |  |
| ≥80p (≥59,311) | 5,017 (18.47) | 13,131 (36.91) |  |
| N/A | 477 (1.76) | 744 (2.09) |  |
| **Body mass index, kg/m^2^, mean (SD)** | 23.41 (3.31) | 23.58 (3.37) | **<0.001** |
| **Systolic blood pressure, mmHg, mean (SD)** | 120.55 (15.94) | 120.64 (16.01) | 0.485 |
| **Diastolic blood pressure, mmHg, mean (SD)** | 75.67 (10.77) | 75.87 (11.00) | **0.023** |
| **Total cholesterol, mg/dL, mean (SD)** | 193.07 (42.55) | 195.01 (44.61) | **<0.001** |
| **Fasting blood glucose, mg/dL, mean (SD)** | 95.20 (26.02) | 97.08 (29.65) | **<0.001** |
| **Current cigarette smoking, N (%)** | 6,360 (23.41) | 7,791 (21.90) | **<0.001** |
| **Current alcohol consumption, N (%)** | 16,097 (59.25) | 20,950 (58.89) | **<0.001** |
| **Mean years of follow-up, mean (SD)** | 7.45 (4.48) | 5.98 (3.98) | **<0.001** |
| **Major cardiovascular events, N (%)^a^** |  |  |  |
| CAD with revascularization | 951 (3.50) | 925 (2.60) | **<0.001** |
| Ischaemic stroke | 1,014 (3.73) | 1,732 (4.87) | **<0.001** |
| Haemorrhagic stroke | 535 (1.97) | 843 (2.37) | **<0.001** |
| **Psychiatric comorbidities, N (%)^a^** |  |  |  |
| Psychotic disorders | 1,078 (3.97) | 3,537 (9.94) | **<0.001** |
| Manic episodes and/or bipolar disorders | 1,751 (6.44) | 6,425 (18.06) | **<0.001** |
| Depressive symptoms and/or disorders | 11,369 (41.84) | 28,436 (79.93) | **<0.001** |
| Anxiety symptoms and/or disorders | 6,239 (22.96) | 17,769 (49.95) | **<0.001** |
| Somatoform disorders | 6,758 (24.87) | 14,357 (40.36) | **<0.001** |
| **Admission due to psychiatric disorder, N (%)^a^** | 2,364 (8.70) | 5,614 (15.78) | **<0.001** |

SD, standard deviation; KRW, Korean Won; N/A, not applicable; CAD, coronary artery disease.

^a^ Numbers and proportions of the participants who had at least one corresponding diagnostic record at the end of follow-up are provided.
